# Adverse events of opioids for cancer-related pain in a resource-limited setting: a cross-sectional study from Sudan

**DOI:** 10.3332/ecancer.2022.1366

**Published:** 2022-03-24

**Authors:** Moawia Mohammed Ali Elhassan, Amal Abdulbagi Abdulfatah Mohammed, Amnah Abdulazeem Omer, Arafa Ahmed Mohammed Azeem, Hiba Mohammed Abdelkfi Mohammed, Isra Elameen Mustafa Ibrahim, Nashwa Abdelaziz Abdelrheem Ahmed

**Affiliations:** 1 Department of Oncology, National Cancer Institute, University of Gezira, PO Box 20, Wad Madani, Gezira State, Sudan; 2 Department of Clinical Pharmacy and Pharmacy Practice, Faculty of Pharmacy, University of Gezira, PO Box 20, Wad Madani, Gezira State, Sudan; a https://orcid.org/0000-0002-0595-5186

**Keywords:** cancer pain, opioids, analgesics, morphine, pain management, adverse events

## Abstract

**Aim:**

Data on the adverse events of opioids for cancer-related pain in Sudanese patients are limited. We conducted this study to evaluate the pattern and severity of adverse events of immediate release morphine, and tramadol used in the treatment of cancer-related pain. A secondary aim was to determine the response rate to opioids for pain control in cancer patients treated at the National Cancer Institute-University of Gezira (NCI-UG), Sudan.

**Methods:**

This descriptive cross-sectional study was conducted at NCI-UG between 12 March 2019 and 12 May 2019. A pre-designed questionnaire was used to collect the clinical data of cancer patients on oral opioids for pain control during the study periods. Chi square test was applied to determine whether there is a significant association between the adverse events and the demographic/clinical variables. *p* value < 0.05 was considered statistically significant in all analyses.

**Results:**

One-hundred and thirteen patients were screened in the study. Of these, three suffered from cognitive dysfunction and ten patients declined to participate in the study. Thus, 100 patients met the criteria for inclusion in this study. Breast cancer was the most frequent diagnosis (17%) followed by colorectal cancer (16%). The majority (91%) of patients had advanced or metastatic disease. The most frequently reported opioids-related adverse events were dry mouth (68%), constipation (61%), sedation (49%), nausea (31%) and vomiting (24%). Most of these symptoms were graded as mild to moderate and no patient had grade IV or V adverse events. Constipation and dry mouth were more frequent among patients received morphine compared to patients received tramadol (*p* value < 0.005). Pain was controlled in 36% of patients, improved in 53% and not controlled in 11% of them.

**Conclusion:**

This study shows a high prevalence of opioids-related adverse events. The majority of the opioids-related adverse events were grade I or grade II. There seem to be differences in the prevalence of opioids-related adverse events between patients receiving tramadol and those treated with morphine. Moreover, suboptimal pain control adds to the burden on already limited health resources. Therefore, the adequacy of cancer pain management in our setting should be systematically evaluated and effective cancer pain management programmes should be developed.

## Background

Pain is one of the most feared and burdensome symptoms experienced by cancer patients, particularly in the advanced and terminal stages of the disease when the prevalence approaches 80% [1, 2]. Cancer related pain greatly affects daily activity and patient quality of life (QOL). Therefore, effective pain control may significantly improve the QOL of these patients, and spare families the feeling of helplessness and despair. In this regard, the World Health Organization (WHO) designed a 3-step ‘analgesic ladder’ practice guidelines to facilitate and standardise pharmacologic cancer pain management. According to the WHO analgesic ladder algorithm, selection of non-opioid analgesics, opioid analgesics and adjuvant analgesic therapy should be individualised, as directed by the intensity of the pain. Treatment of mild pain (step I) consists of using non-opioid analgesics, which include acetaminophen, aspirin, non-steroidal anti-inflammatory drugs (NSAIDs). Weak opioids such as codeine or Tramadol are recommended in the treatment of moderate pain (step II). Morphine is considered the drug of first choice for treatment of moderate to severe cancer pain (step III) [3].

The goals of pain management are to reduce pain and improve QOL while limiting excessive adverse events. Opioids-related adverse events can reduce QOL and limit opioids dosage which leads to ineffective cancer pain management [4]. Common opioids related adverse events include nausea, constipation, dry mouth, sedation and confusion [4–6]. Many of these adverse events are difficult to distinguish from disease progression or other pathological causes.

Sudan is a low-income country in sub-Saharan Africa with a population of approximately 40 million [2]. The majority of the population lives in rural settings. The health services in Sudan are organised at three levels: primary, secondary and tertiary. Khartoum Oncology Hospital in Khartoum and the National Cancer Institute, University of Gezira (NCI-UG), which is located at Wad Madani, the capital of Gezira State, are the only two specialised cancer centres providing both chemotherapy and radiotherapy services for the entire country. More recently, chemotherapy units attached to eight public state hospitals have been established by the government. The national policy adopted by the Federal Ministry of Health is to provide free of charge health care services for cancer patients at public cancer treatment centres.

A previous single institution study conducted in Sudan showed that uncontrolled pain was the most frequent symptom that prompted patients with cancer to attend the outpatient unit without an appointment [7]. Data on adequacy of cancer pain management as well as adverse events related to pain medications in our limited resource settings are limited. In Sudan, where access to palliative care is limited and there is no hospice care, the opioids medications that are available for patients with cancer are tramadol and immediate-release morphine. Some opioids-related adverse events appear to be underestimated. No previous study about the pattern and severity of opioids related adverse events has been conducted in Sudan. Thus, the current study was conducted to assess the pattern and severity of oral opioids related adverse events among cancer patients at NCI-UG, Sudan.

## Methods

### Setting

NCI-UG specialises in the diagnosis and treatment of cancer patients. It is located in Wad Medani city, the capital of Gezira state, which serves the whole Gezira state and nearby states. Radiotherapy, chemotherapy and palliative care are cancer treatment modalities available at the NCI-UG.

### Study design

This prospective cross-sectional study was conducted to evaluate the pattern and severity of oral opioids related adverse events among cancer patients at the NCI-UG between 12 March 2019 and 12 May 2019. A secondary aim was to determine response rate to opioids for pain control in this cohort of patients.

### Inclusion and exclusion criteria

Cancer patients (>16 years old) on oral opioids (short acting morphine or tramadol) for at least 1 week were eligible to be included in the study. Immediate release morphine and tramadol are the only available opioids in our setting. Patients unable to answer the interviewer or patients who refuse to participate in the study were excluded.

### Data collection

A predesigned paper-based questionnaire containing eight parts was developed by the principal investigator. The questionnaire was submitted to a panel of three experts to review the content accuracy and internal validity. It was then piloted on ten patients and modifications were made according to the suggestions. The patients were interviewed by the researchers and a questionnaire was filled out based on patients’ responses to the questions. The data collected included: demographics data (age, sex, marital status and residence), clinicopathological variables (primary cancer site, stage and co-morbidities), types of pain and severity according to a 5-point numerical scale (1–2 represent mild, 3 moderate and 4–5 for severe pain), types of opioid analgesics, response to pain medications and opioids related adverse events according to the Common Terminology Criteria for Adverse Events Version 4.0, National Institute of Health, National Cancer Institute. Consent was obtained from participants prior to their inclusion in the study.

### Data analysis

Data were entered into and analysed using Excel software and SPSS version 24. Categorical variables are presented as frequencies and percentages. Chi square test was applied to determine whether there is a significant association between the adverse events and the demographic/clinical variables. *p* value of less than 0.05 was considered statistically significant in all analyses.

### Ethical approval

The study was approved by the ethics committee of the NCI-UG, Sudan. Data were collected anonymously.

## Results

One-hundred and thirteen patients were screened in the study. Of these, three suffered from cognitive dysfunction, ten patients declined to participate in the study. Thus, 100 patients met the criteria for inclusion in this study. The majority of patients were female (65%). Breast cancer was the most frequent diagnosis (17%) followed by colorectal cancer (16%). The majority (91%) of patients had advanced or metastatic disease. The general characteristics of the study population are presented in Table 1.

Of the included patients, 45% suffered from somatic pain, 45% suffered from visceral pain and 10% suffered from neuropathic pain. As shown in Table 2, most (54%) of our study population received tramadol and 46% received short acting oral morphine. Pain was controlled in 36% of patients, improved in 53% and not controlled in 11% of them.

The most frequently reported opioids-related adverse events are shown in Table 3. Dry mouth was the most prevalent adverse event (68%), followed closely by constipation (61%), decreased level of consciences (49%), nausea (31%) and vomiting (24%). Less frequently reported symptoms were dizziness, pruritis, confusion and delirium. Most of these symptoms were graded as mild to moderate as shown in Table 3. Constipation was usually intermittent (37%) or persistent (18%).

**Figure 1.**
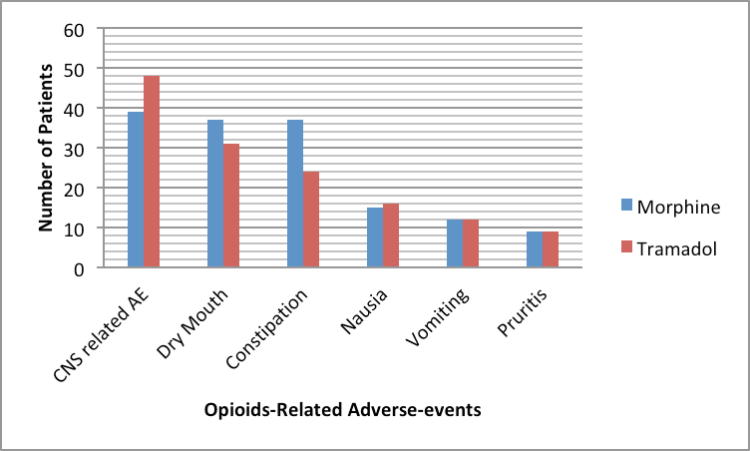
Distribution of adverse events according to the types of opioids. Note: Abbreviations: CNS, Central nervous system; AE, Adverse events.

Figure 1 shows the frequency of opioids-related adverse events in patients treated with morphine and tramadol. Association between the three most frequent opioids-related adverse events and the demographic/clinical variables among our study population was shown in Tables 4–6. There were no statistically significant differences were observed in the frequency of the three most frequent opioids-related adverse events across strata of selected patients’ demographic and clinical characteristics. We observed significant differences in frequency of constipation and dry mouth according to the type of opioids received by cancer patients. Constipation and dry mouth were more frequent among patients received morphine compared to patients received tramadol (*p* value < 0.005). Thirty patients in the tramadol group (55.6%) and 9 patients (19.6%) in the morphine group were free of constipation during the study period. Among patients who did not reported opioids-related dry mouth, 23 patients were in the tramadol group (42%) and 9 patients (19.6%) in the morphine group. Although not statistically significant, adverse events of opioids on the central nervous systems (CNS) are more frequent among patients treated with tramadol com pared to patients received morphine (Table 4). The nausea, vomiting, dizziness, pruritis had similar frequency in patients with morphine and tramadol.

## Discussion

In Sudan, the majority of cancer patients have locally advanced or metastatic disease at presentation [8–13]. Currently available data from Sudan suggest that 90% of the terminally ill cancer patients suffered from uncontrolled pain [14]. Moreover, uncontrolled pain is among the most frequent symptoms that prompted unplanned visit to the outpatient oncology unit in our limited resource setting [7]. In the current study, pain was controlled in only 36% of cases on opioids. These clearly suggest that the deleterious effects of uncontrolled pain on cancer patients and the already limited health resources are of significant proportions. Lack of knowledge among physician and patients, lack of adequate supply of opioids as well as limited access to palliative care services are the main causes of inadequate pain control in countries with limited resources [15]. Besides this, opioids-related adverse events remain the most important barriers that interfere with adherence to opioid analgesics [16].

In Sudan, opioid analgesics are available free of charge for patients with malignant disorders in cancer treatment centres. Opioid prescriptions are dispensed for a maximum 1-month supply. Therefore, cancer patients on opioid analgesics or their caregivers need to travel from remote areas every month to collect pain medications from cancer treatment centres. The cost of travel represents a huge burden for the patients and their families. Thus, implementing dedicated pain clinics for providing follow-up services and pain medications for cancer patients at primary and secondary health care centres may help reduce unnecessary visits to cancer treatment centres.

Understanding the incidence and severity of opioids-related side effects helps the clinician frame an optimal management plan. Fear of opioids-related adverse events may result in some withholding of opioids administration by the treating physician, often aggravating patients’ pain. To the authors’ knowledge, this is the first study providing an overview of opioids-related adverse events for cancer-related pain in Sudan. In general, we found that the occurrence rates of opioids-related side effects (of any grade) were high. The majority of adverse events reported rated as mild to moderate. Constipation, dry mouth and alteration level of consciousness seem to be of particular impact because of high prevalence.

In this study, dry mouth was the most frequent (68%) opioids-related adverse event. We found patients who received tramadol experienced significantly less dry mouth compared to those who received morphine. Previous studies showed a large variation (1%–94%) in the occurrence of dry mouth [17, 18]. Studies with low incidence rates documented only opioids-related adverse events spontaneously reported by patients without systematic assessment [19, 20].

Constipation is estimated to occur in 25%–50% of cancer patients. It is frequently related to opioids analgesics in patients with advanced cancer [21]. However, it can also be related to the patient’s condition and co-morbidity. In our study, constipation occurred in approximately two-thirds of cases. It is necessary to undertake preventive measures for reducing the occurrence of opioids-related constipation which would ensure that the benefits of pain treatment are not threatened by the intensity of constipation. In our setting, knowledge and attitudes of physicians about preventive measures for reducing the occurrence of opioids-related constipation and patients’ adherence to these preventive measures are unknown. Therefore, further studies are warranted to assess perceived barriers and preventive measures for reducing the occurrence of opioids-related constipation among health care providers.

Adverse events of opioids on the CNS are common. In this study, sedation was the most frequent (49%) adverse effect of opioids on CNS followed by drowsiness (13%) and delirium (8%). The incidence of sedation, drowsiness and delirium in patients with advanced or metastatic cancer is high. Moreover, its causes are often multiple and can be attributed to opioids, other medicines and the disease itself. Therefore, it is difficult to define how much of the sedation, drowsiness and delirium seen at the end of life can be attributed to opioids and how much to the other causes. Sedation caused by opioids may be reduced by decreasing the dose or by rotation to alternative opioids [6, 22]. In the palliative care setting, haloperidol is the most commonly prescribed medication for the relief of agitated delirium [23, 24].

The incidence of opioids-induced nausea and vomiting is estimated to be 10%–40% [25]. In this study, the reported rate of occurrence of nausea and vomiting was 31% and 24%, respectively. Before treatment of opioids-induced nausea is initiated, readily reversible co-morbidities such as hypercalcaemia and raised intracranial pressure should be addressed. In a large multinational trial of patients requiring treatment with opioid analgesics for pain management, 1 dose of 8 mg or 16 mg of intravenous ondansetron was effective in controlling nausea and vomiting in this patient population [26]. Combined blockade of dopamine and serotonin receptors by haloperidol and ondansetron, respectively, is sometimes required to relieve intractable opioids-induced nausea and vomiting.

Pruritus (itch) is an occasional side effect of opioids use. In this study, pruritus was reported by 18% of our study population. The mechanism underlying the pruritogenic effects of opioids is still not completely understood [5]. Despite controversy about the role of histamine in opioid-induced pruritus, antihistamines are still commonly used as first-line treatment. Reducing the opioids dose has also been suggested [27].

In the current study, we examined possible differences in the prevalence of opioids-related adverse events between patients receiving tramadol and those treated with morphine. The non-opioids characteristics of tramadol may explain the difference in the spectrum of side effects compared to morphine. Tramadol treatment resulted in statistically significant lesser constipation and dry mouth than morphine. Although not statistically significant, CNS related adverse events appear to occur more frequently with tramadol than morphine. The difference in the spectrum of side effects reported in this study should be confirmed in a prospective randomised controlled trial. Moreover, further studies are required to compare the difference in intensity of side effects of tramadol and morphine in patients with cancer pain in our limited resource setting.

The current study is a single institution’s data; we therefore cannot make conclusions for all Sudan and our findings should be generalised with the necessary caution. However, the NCI-UG is the only referral oncology hospital in central Sudan; hence, the current data provide the best indicator of the pattern and severity of oral opioids-related adverse events among cancer patients within this region.

## Conclusion

This study shows a high prevalence of opioids-related adverse events. These adverse events could be obstacles that hinder pain therapy or cause its termination. Therefore, it is necessary to undertake preventive measures for reducing preventable opioids-related adverse events such as constipation and dryness of mouth which would ensure that the benefits of pain treatment are not threatened by the intensity of negative effects. Moreover, uncontrolled pain adds to the burden on already limited health resources. Therefore, the adequacy of cancer pain management in our setting should be systematically evaluated and effective cancer pain management programmes tailored to our limited resources setting should be developed. Further research should focus on exploring the effect of opioids-related adverse events on adherence to pain medications.

## Authors’ contributions

M.M.A.E. was the project leader. All authors contributed to the design and implementation of the research, analysis of the results and writing of the manuscript.

## Conflicts of interest and funding

No funding was received for this study and none of the authors has any conflicts of interest relevant to this work.

## Figures and Tables

**Table 1.** The general characteristics of the study population (*n = 100*).

Demographics	Characteristics	Frequency (%)
**Gender**	Male	35
	Female	65
**Age**	18–27	8
	28–37	12
	38–47	24
	48–57	24
	58–67	14
	˃67	18
**Residence**	Rural	79
	Urban	21
**Marital status**	Married	70
	Single	30
**Co-morbidities**	No	76
	DM ***<AQ: Please spell out DM>***	11
	HTN ***<AQ: Please spell out HTN>***	11
	IHD ***<AQ: Please spell out IHD>***	2
**Types of cancer**	Breast	18
	Colorectal	17
	Head and neck	10
	Haematological malignancies	7
	Prostate	7
	Ovary	5
	Lymphoma	5
	Other	31
**Stages of disease**	1	1
	2	1
	3	35
	4	56
	Unknown	7 (7%)

DM, Diabetes Mellitus; HTN, Hypertension; IHD, Ischemic Heart Disease

**Table 2.** Types and doses of opioids.

Types of opioids	Dose	Number of patients
Morphine		
	5 mg 4 hourly	10
	10 mg 4 hourly	9
	15 mg 4 hourly	8
	20 mg 4 hourly	6
	30 mg 4 hourly	5
	40 mg 4 hourly	8
Tramadol		
	50 mg 6 hourly	53
	100 mg 6 hourly	1

**Table 3.** Frequencies and severity grades of opioids-related adverse events among cancer patients (*n* = 100) treated at the NCI-UG, Sudan.

Opioids-related	Grade of the adverse events					Total	Proportions of totals (95% CI)
adverse events	I	II	III	IV	V		
Dry mouth	40	28	0	0	0	68	0.68 (0.59–0.77)
Constipation	37	18	6	0	0	61	0.61 (0.51–0.71)
Sedation	14	29	6	0	0	49	0.49 (0.39–0.59)
Nausea	14	17	1	0	0	31	0.31 (0.22–0.40)
Vomiting	13	8	3	0	0	24	0.24 (0.16–0.32)
Pruritus	14	4	0	0	0	18	0.18 (0.11–0.26)
Dizziness	9	5	3	0	0	17	0.17 (0.10–0.24)
Drowsiness	8	3	2	0	0	13	0.13 (0.06–0.19)
Delirium	7	1	0	0	0	8	0.08 (0.03–0.13)

A grading scale: I: mild; asymptomatic or mild symptoms; clinical or diagnostic observation only; intervention not indicated. II: moderate; minimal, local or noninvasive intervention indicated; limiting age-appropriate instrumental activities of daily living. III: severe or medically significant but not immediately life-threatening; hospitalisation or prolongation of hospitalisation indicated; disabling; limiting self-care. Grade 4: life-threatening consequences; urgent intervention indicated. Grade 5: death related to adverse event

**Table 4.** Association between the CNS related adverse events and the demographic/clinical variables among cancer patients (*n* = 100) treated with opioids for cancer-related pain at the NCI-UG, Sudan.

Demographic/clinical variables		CNS related adverse events		Total	*p* value
		No	Yes		
Category	Sub-category	*N* (%)	*N* (%)	*N* (%)	
Sex	Male	5 (14.3)	30 (85.7)	35 (100)	0.78
	Female	8 (12.3)	57 (87.7)	65 (100)	
Age group	<50 years	8 (18.2)	36 (81.8)	44 (100	0.17
	≥50 years	5 (8.9)	51 (91.1)	56 (100)	
Residence	Rural	11 (13.9)	68 (86.1)	79 (100)	0.59
	Urban	2 (9.5)	19 (90.5)	21 (100)	
Marital status	Married	7 (10.0)	63 (90)	70 (100)	0.17
	Single	6 (20.0)	24 (80)	30 (100)	
Types of pain	Visceral	5 (11.1)	40 (88.9)	45 (100	0.75
	Somatic	6 (13.3)	39 (86.7)	45 (100)	
	Neuropathic	2 (20.0)	8 (80.0)	10 (100)	
Opioids type	Tramadol	6 (11.1)	48 (88.9)	54 (100)	0.54
	Morphine	7 (15.2)	39 (84.8)	46 (100)	

Note: Abbreviations: *N*, Number of cases; CNS, Central nervous system; NCI-UG, The National Cancer Institute-University of Gezira

**Table 5.** Association between the constipation and the demographic/clinical variables among cancer patients (*n* = 100) treated with opioids for cancer-related pain at the NCI-UG, Sudan.

Demographic/clinical variables		Constipation		Total	*p* value
		No	Yes		
Category	Sub-category	*N* (%)	*N* (%)	*N* (%)	
Sex	Male	15 (42.9)	20 (57.1)	35 (100)	0.56
	Female	24 (36.9)	41 (63.1)	65 (100)	
Age group	<50 years	19 (43.2)	25 (56.8)	44 (100)	0.45
	≥50 years	20 (35.7)	36 (64)	56 (100)	
Residence	Rural	32 (40.5)	47 (59.5)	79 (100)	0.55
	Urban	7 (33.3)	14 (66.7)	21 (100)	
Marital status	Married	24 (34.3)	46 (65.7)	70 (100)	0.14
	Single	15 (50.0)	15 (50)	30 (100)	
Types of pain	Visceral	18 (40.0)	27 (60.0)	45 (100)	0.83
	Somatic	18 (40.0)	27 (60.0)	45 (100)	
	Neuropathic	3 (30.0)	7 (70.0)	10 (100)	
Opioids type	Tramadol	30 (55.6)	24 (44.4)	54 (100)	0.001
	Morphine	9 (19.6)	37 (80.4)	46 (100)	

Note: Abbreviations: *N*, Number of cases; NCI-UG, The National Cancer Institute-University of Gezira

**Table 6.** Association between the dry mouth and the demographic/clinical variables among cancer patients (*n* = 100) treated with opioids for cancer-related pain at the NCI-UG, Sudan.

Demographic/clinical variables		Dry mouth		Total	*p* value
		No	Yes		
Category	Sub-category	*N* (%)	*N* (%)	*N* (%)	
Sex	Male	16 (45.7)	19 (54.3)	35 (100)	0.03
	Female	16 (24.6)	49 (75.4)	65 (100)	
Age group	<50 years	13 (29.5)	31 (70.5)	44 (100)	0.64
	≥50 years	19 (33.9)	37 (66.1)	56 (100)	
Residence	Rural	27 (34.2)	52 (65.8)	79 (100)	0.36
	Urban	5 (23.8)	16 (76.2)	21 (100)	
Marital status	Married	21 (30.0)	49 (70.0)	70 (100)	0.51
	Single	11 (36.7)	19 (63.3)	30 (100)	
Types of pain	Visceral	19 (42.2)	26 (57.8)	45 (100)	0.13
	Somatic	10 (22.2)	35 (77.8)	45 (100)	
	Neuropathic	3 (30.0)	7 (70.0)	10 (100)	
Opioids type	Tramadol	23 (42.6)	31 (57.4)	54 (100)	0.01
	Morphine	9 (19.6)	37 (80.4)	46 (100)	

Note: Abbreviations: *N*, Number of cases; NCI-UG, The National Cancer Institute-University of Gezira
